# Gα_i_ is required for carvedilol-induced β_1_ adrenergic receptor β-arrestin biased signaling

**DOI:** 10.1038/s41467-017-01855-z

**Published:** 2017-11-22

**Authors:** Jialu Wang, Kenji Hanada, Dean P. Staus, Michael A. Makara, Giri Raj Dahal, Qiang Chen, Andrea Ahles, Stefan Engelhardt, Howard A. Rockman

**Affiliations:** 1 Department of Cell Biology, Duke University Medical Center, Durham, NC 27710 USA; 2 Department of Medicine, Duke University Medical Center, Durham, NC 27710 USA; 3 Institute of Pharmacology and Toxicology, Technical University of Munich, Munich, 80802 Germany; 4 German Center for Cardiovascular Research (DZHK), Partner Site Munich Heart Alliance, Munich, 80802 Germany; 5 Departments of Molecular Genetics and Microbiology, Duke University Medical Center, Durham, NC 27710 USA

## Abstract

The β_1_ adrenergic receptor (β_1_AR) is recognized as a classical Gα_s_-coupled receptor. Agonist binding not only initiates G protein-mediated signaling but also signaling through the multifunctional adapter protein β-arrestin. Some βAR ligands, such as carvedilol, stimulate βAR signaling preferentially through β-arrestin, a concept known as β-arrestin-biased agonism. Here, we identify a signaling mechanism, unlike that previously known for any Gα_s_-coupled receptor, whereby carvedilol induces the transition of the β_1_AR from a classical Gα_s_-coupled receptor to a Gα_i_-coupled receptor stabilizing a distinct receptor conformation to initiate β-arrestin-mediated signaling. Recruitment of Gα_i_ is not induced by any other βAR ligand screened, nor is it required for β-arrestin-bias activated by the β_2_AR subtype of the βAR family. Our findings demonstrate a previously unrecognized role for Gα_i_ in β_1_AR signaling and suggest that the concept of β-arrestin-bias may need to be refined to incorporate the selective bias of receptors towards distinct G protein subtypes.

## Abstract (web summary)

β1 and β2 adrenergic receptors (ARs) are the predominant G protein-coupled receptor subtypes expressed in mammalian hearts. Here the authors describe a signaling mechanism where the biased agonist carvedilol converts β1AR from a classical Gα_s_-coupled to a Gα_i_-coupled receptor to mediate β-arrestin-dependent signaling.

## Introduction

G protein-coupled receptors (GPCRs) represent the largest and the most versatile family of cell surface receptors^1^. Members of this receptor family translate diverse extracellular cues to intracellular responses, and are commonly targeted for medicinal therapeutics^2, 3^. One of the most commonly used therapeutic agents in medicine are ligands that target β adrenergic receptors (βARs) because they regulate many important physiological processes involved in the regulation of cardiovascular and pulmonary function^4^.

GPCRs selectively couple to different heterotrimeric G protein complexes (Gαβγ) that are classified into four families based on their α-subunits: Gα_stimulatory_ (Gα_s_), Gα_inhibitory/olfactory_ (Gα_i/o_), Gα_q/11_, and Gα_12/13_^5^. Among the different G protein subtypes, βARs primarily transmit signals through Gα_s_^6^. In the classical paradigm of βAR signaling, receptors exist in two distinct conformational states: active or inactive. Agonist binding stabilizes an active βAR conformation that promotes coupling with heterotrimeric G proteins, triggering guanine nucleotide exchange of Gα_s_ and its dissociation from the Gβγ subunits, leading to the activation of adenylyl cyclase and triggering second messenger cyclic AMP signaling^7, 8^. Subsequent to agonist binding, activated βARs are phosphorylated by G protein-coupled receptor kinases (GRKs) leading to recruitment of the multifunctional β-arrestins (β-arrestin1 and β-arrestin2) and inhibition of further G protein coupling, a process termed desensitization^8^. It is now appreciated that β-arrestins also act as signal transducers in their own right^7^ to stimulate a distinct array of signaling and cellular responses, such as transactivation of the epidermal growth factor receptor (EGFR)^9, 10^, induction of extracellular signal-regulated kinase (ERK)^10–13^, and activation of Ca^2+^/calmodulin kinase II (CaMKII)^14^. Current data suggest a much greater complexity of GPCR signaling than the two-state (active or inactive) model whereby multiple receptor conformations can exist, each with a different affinity for its transducer, resulting in the activation of distinct cellular signaling pathways^15–17^. Whereas balanced ligands, such as isoproterenol, stabilize βAR conformations signal with equal efficacy through G proteins and β-arrestins, some ligands stabilize conformations that selectively recruit only one of the transducers to stimulate a specific subset of cellular signals, a process termed “biased agonism”^18, 19^. As biased ligands may be capable of selectively activating beneficial signaling while simultaneously blocking untoward receptor activated pathways^20^, understanding mechanisms of biased agonism can have important implications for drug discovery targeting GPCRs.

The β-blocker carvedilol is a β-arrestin-biased βAR ligand that preferentially activates β-arrestin-mediated pathways while having inverse agonism towards Gα_s_ signaling^7, 10, 19, 21^. To date, the prevailing mechanistic concept of β-arrestin-bias for the Gα_s_-coupled β_1_AR is ligand-stimulated activation of β-arrestin in the absence of G protein coupling. However, recently it has been demonstrated for the angiotensin II type 1 receptor that the weak β-arrestin-biased agonist, [^1^Sar^4^Ile^8^Ile]-angiotensin II, is capable of activating both Gα_q_ and Gα_i_^22^, indicating a possible role of G proteins in β-arrestin-mediated signaling. Moreover, recent biophysical work suggests that both G protein and β-arrestin can simultaneously interact with an activated GPCR to form super complexes^23^, raising the possibility that association of β-arrestin with the receptor may not preclude interaction with a G protein. Here, we set out to test whether G protein coupling is a critical component of β-arrestin-biased βAR signaling. Our findings show that carvedilol, unique among other βAR agonists or antagonists tested, selectively promotes the recruitment of Gα_i_ to β_1_ARs to initiate β-arrestin-biased signaling. These data underscore the complexity of β-arrestin-biased agonism and have important implications for identifying new therapeutic agents to selectively target β-arrestin-biased signaling.

## Results

### Gα_i_ is required for carvedilol-induced β_1_AR-mediated ERK

**Fig. 1:**
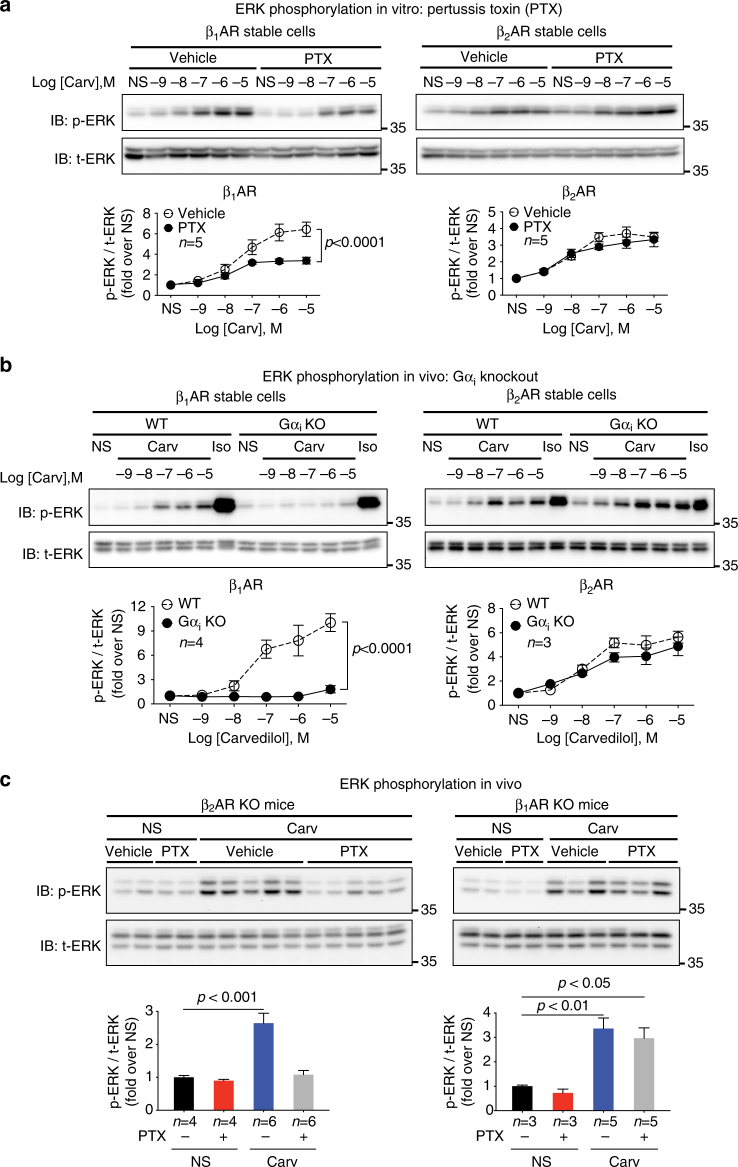
Gα_i_ is required for the carvedilol-induced β_1_AR-mediated ERK phosphorylation both in vitro and in vivo. **a** Effect of PTX on carvedilol-induced βAR-mediated ERK phosphorylation in HEK293 cells. HEK293 cells stably expressing FLAG-tagged β_1_ARs or β_2_ARs were pretreated with vehicle or 200 ng per ml PTX for 16 h, then stimulated with indicated concentration of carvedilol for 5 min. Carvedilol induced ERK phosphorylation in both β_1_AR or β_2_AR stable cells in dose-dependent manner. The response in β_1_AR stable cells was blocked by PTX, whereas that in β_2_AR stable cells was PTX insensitive. **b** Effect of Gα_i_ knockout on βAR-mediated ERK phosphorylation in HEK293 cells. The Gα_i_ expression in β_1_AR or β_2_AR stable cells was depleted with CRISPR–Cas9 gene editing. Compared with wild-type β_1_AR stable cells, the carvedilol-induced ERK phosphorylation in Gα_i_ knockout β_1_AR stable cells was diminished. In comparison, the response in β_2_AR stable cells was not affected. **c** Effect of PTX on carvedilol-stimulated ERK phosphorylation in Langendorff perfused hearts from β_2_AR knockout mice or β_1_AR knockout mice. Mice were pretreated with vehicle or 25 μg per kg PTX through intraperitoneal injection. 48 h after injection, mice hearts were excised and perfused with vehicle or 10 μM carvedilol for 10 min. PTX diminished the carvedilol-induced ERK phosphorylation in hearts from β_2_AR knockout mice, but not β_1_AR knockout mice. Data represent the mean ± SEM for *n* independent experiments (**a**, **b**) or *n* animals (**c**) as marked on the figure. Statistical significance vs. control was assessed using two-way ANOVA (**a**, **b**) or one-way ANOVA (**c**) with Bonferroni correction. NS no stimulation; Carv carvedilol; Iso isoproterenol; p-ERK phosphorylated ERK; t-ERK total ERK; WT wild type; KO knockout

Previous studies have demonstrated that carvedilol induces βAR-mediated ERK phosphorylation in a Gα_s_-independent, β-arrestin-dependent manner^10, 21^. To determine whether Gα_i_ is required for carvedilol-stimulated βAR signaling, we tested the effect of the Gα_i_ inhibitor pertussis toxin (PTX) on carvedilol-stimulated ERK phosphorylation in HEK293 cells stably expressing FLAG-tagged β_1_AR or β_2_AR. PTX catalyzes the ADP-ribosylation of Gα_i_ and prevents Gα_i_ coupling to ligand bound receptors. In β_1_AR stable cells, carvedilol dose dependently increased ERK phosphorylation, which was significantly diminished by pretreatment with the Gα_i_ inhibitor PTX (Fig. 1a, Supplementary Fig. 1a). In contrast, PTX had no effect on the carvedilol-induced β_2_AR-mediated ERK phosphorylation (Fig. 1a, Supplementary Fig. 1a). These observations suggest that Gα_i_ is needed for carvedilol-induced β_1_AR, but not β_2_AR signaling.

To further delineate the role of Gα_i_ in carvedilol-induced βAR signaling, we measured the level of ERK activation in β_1_AR or β_2_AR stable cells after removing Gα_i_ using CRISPR/Cas9 gene editing. All three subtypes of Gα_i_ (Gα_i1_, Gα_i2_, and Gα_i3_) were depleted with their specifically targeted guide RNAs (Supplementary Fig. 1b). Gα_i_ depletion markedly blocked carvedilol-induced ERK phosphorylation in β_1_AR stable cells, while it had no effect in β_2_AR stable cells (Fig. 1b). The absence of Gα_i_ was considerably more robust in abrogating carvedilol-stimulated ERK phosphorylation compared to that observed with PTX treatment (Fig. 1a).

We then determined if a similar signaling mechanism is involved in heart tissue by measuring ERK phosphorylation in Langendorff perfused mouse hearts following carvedilol stimulation. To study the specific effect of carvedilol on the β_1_AR, we used previously generated β_2_AR knockout mice^24^. Carvedilol perfusion robustly stimulated ERK phosphorylation in hearts of β_2_AR knockout mice, which was entirely abrogated in hearts of PTX-pretreated mice (Fig. 1c). In contrast, in β_1_AR knockout mice^25^ while carvedilol robustly induced ERK phosphorylation by activating the β_2_AR, PTX pretreatment was unable to block ERK activation (Fig. 1c). These data are consistent with our in vitro data and indicate a previously unrecognized, βAR subtype specific, requirement for Gα_i_ in carvedilol-induced β_1_AR signaling.

### Carvedilol-induced β_1_AR conformational change requires Gα_i_

**Fig. 2:**
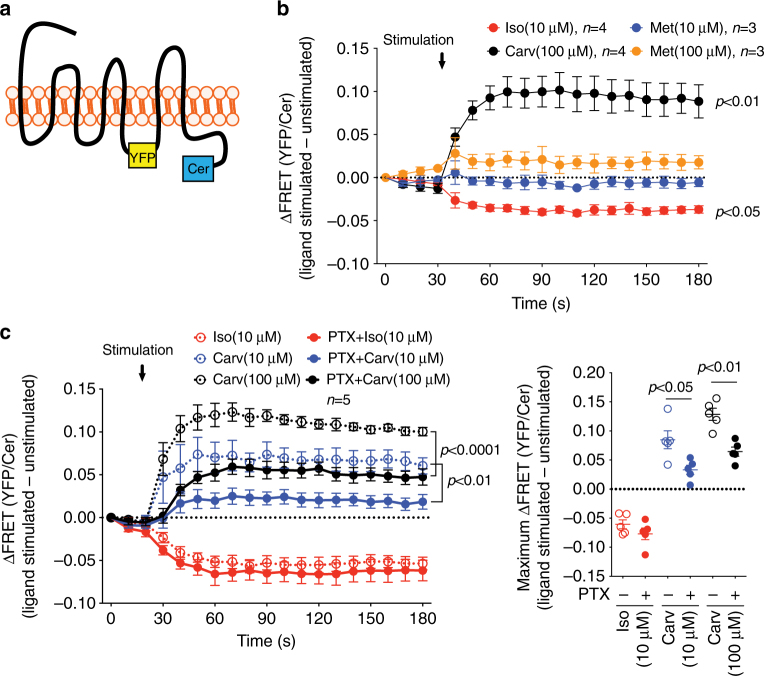
Gα_i_ is required for carvedilol-induced β_1_AR conformation change. **a** In the FRET-based β_1_AR conformation sensor, YFP and Cerulean (Cer) are inserted in the third intracellular loop and the C-tail of β_1_AR respectively. **b** Ligand-induced changes of the FRET ratio in HEK293 cells stably expressing β_1_AR FRET sensor. The stable cells were stimulated with 100 μM carvedilol, 10 μM isoproterenol, 10 μM or 100 μM β_1_AR antagonist metoprolol, while FRET was monitored in real-time as the emission ratio of YFP to Cer. Carvedilol stimulation increased the FRET ratio, while isoproterenol decreased it, demonstrating the distinct β_1_AR conformations induced by these two ligands. Metoprolol had no effect on the FRET ratio. **c** Effect of PTX on ligand-induced FRET ratio change. Cells were pretreated with vehicle or 200 ng per ml PTX for 16 h before ligand stimulation. PTX blocked carvedilol-induced change, while having no effect on the isoproterenol response, suggesting that Gα_i_ is required to stabilize the carvedilol-induced β_1_AR conformation. Data represent the mean ± SEM for *n* independent experiments as marked on the figure. Statistical significance vs. unstimulated cells was assessed using one-way ANOVA with Bonferroni correction (**b**); statistical significance between PTX-pretreated and non-pretreated cells was assessed using two-way ANOVA with Bonferroni correction (**c**, left panel), or two-tailed paired Student’s *t*-test (**c**, right panel)

Different ligands for the same receptor stabilize unique conformational states promoting coupling to selective signal transducers and activation of distinct downstream signaling pathways^7, 20^. Since we showed that Gα_i_ is required for carvedilol-induced β_1_AR signaling, we tested whether it allosterically stabilizes a unique carvedilol-bound β_1_AR conformation. We utilized a fluorescence resonance energy transfer (FRET)-based β_1_AR conformational sensor in which Cerulean (Cer) and YFP are inserted in the C-terminus and third intracellular loop of the receptor, respectively (Fig. 2a)^26^. Agonist-induced β_1_AR activation is represented by the loss of FRET, i.e., decrease of YFP/Cer ratio^26^. To test whether Gα_i_ stabilizes a carvedilol-induced β_1_AR conformation, HEK293 cells stably expressing the β_1_AR FRET sensor were pretreated with vehicle or PTX, then stimulated with the balanced agonist isoproterenol or the β-arrestin-biased agonist carvedilol while monitoring the FRET ratio in real time. We found that compared to isoproterenol which caused a decrease in the FRET ratio, carvedilol induced a directional opposite response to the FRET signal, whereas the β_1_AR antagonist metoprolol showed no effect (Fig. 2b). Importantly, pretreatment with PTX significantly diminished the carvedilol-induced FRET ratio without any effect on the isoproterenol stimulated FRET-based receptor biosensor (Fig. 2c). Lastly, PTX alone did not affect the FRET ratio (Supplementary Fig. 2). These data demonstrate the β_1_AR adopts a distinct conformational state when bound to isoproterenol compared to carvedilol and that Gα_i_ is needed to stabilize the carvedilol-bound β_1_AR conformation.

### Carvedilol selectively promotes Gα_i_ recruitment to β_1_ARs

**Fig. 3:**
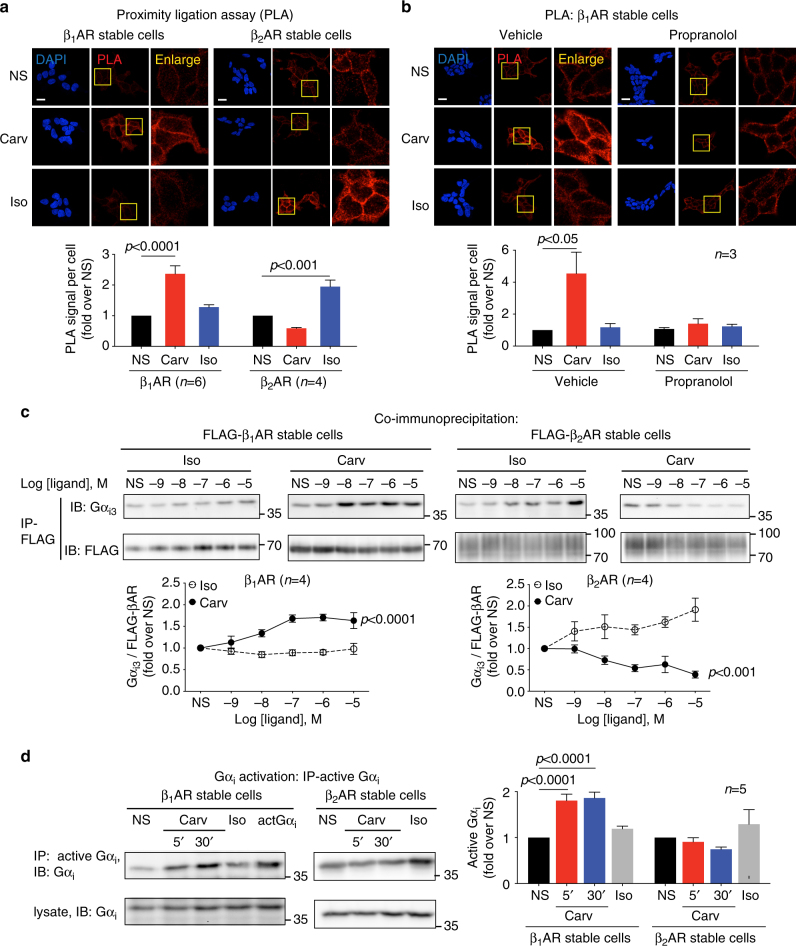
Carvedilol promotes Gα_i_ recruitment and activation in β_1_AR stable cells, but not in β_2_AR stable cells. HEK293 cells stably expressing FLAG-tagged β_1_ARs or β_2_ARs were stimulated with 10 μM carvedilol or 10 μM isoproterenol for 5 min. **a** In proximity ligation assay (PLA), cells were immuno-stained with Gα_i_ antibody raised in mouse and β_1_AR (or β_2_AR) antibody raised in rabbit. The red PLA signal represents the protein interactions of Gα_i_ and β_1_AR (or β_2_AR). The area in yellow squares are enlarged for better view. Carvedilol promoted Gα_i_ recruitment to β_1_ARs, but not to β_2_ARs. Scale bar = 20 μm. **b** β_1_AR stable cells were pretreated with vehicle or 10 μM propranolol for 30 min. The βAR antagonist propranolol blocked the carvedilol response, suggesting that β_1_AR–Gα_i_ coupling is induced by the binding of carvedilol to the β_1_AR orthosteric binding pocket. Scale bar = 20 μm. **c** The effect of carvedilol and isoproterenol on Gα_i_ recruitment was confirmed with co-immunoprecipitation assays. FLAG-tagged β_1_ARs or β_2_ARs were immunoprecipitated with anti-FLAG M2 beads, and Gα_i3_ was detected with its specific antibody by western blot. **d** Carvedilol specifically activated Gα_i_ in β_1_AR stable cells. Cells were transfected with Gα_i2_. 48 h after transfection, cells were treated with 10 μM carvedilol for 5 min or 30 min, or 10 μM isoproterenol for 5 min. Activated Gα_i_ was immunoprecipitated with an antibody specifically recognizing the active form of Gα_i_, and immunoblotted with an Gα_i_ antibody. In the middle lane marked as actGα_i_, cells were transfected with constitutively active Gα_i2_, serving as positive control. Carvedilol stimulation activated Gα_i_ in β_1_AR stable cells, but not in β_2_AR stable cells. Data represent the mean ± SEM for n independent experiments as marked on the figure. Statistical significance vs. unstimulated cells (NS) was assessed using one-way ANOVA with Bonferroni correction

To determine the mechanism of how Gα_i_ is involved in carvedilol-induced β_1_AR signaling, we measured ligand-promoted Gα_i_ recruitment to βARs with an in situ proximity ligation assay (PLA), a confocal-microscopy based assay that allows direct visualization and quantification of protein–protein interactions. Using HEK293 cells stably expressing β_1_ARs, we show an over twofold increase in the PLA signal after carvedilol treatment, indicating recruitment of Gα_i_ to the β_1_AR (Fig. 3a). In contrast, carvedilol had no effect on the recruitment of Gα_i_ to β_2_ARs, but Gα_i_ was robustly recruited by isoproterenol consistent with the known process of G protein switching for β_2_ARs^27^ (Fig. 3a). Importantly, pretreatment with the βAR antagonist propranolol blocked the carvedilol-induced Gα_i_ recruitment to β_1_ARs (Fig. 3b), indicating the recruitment is dependent on ligand interaction with the β_1_AR orthosteric binding pocket. To further demonstrate recruitment of Gα_i_ to carvedilol-stimulated β_1_AR, we also performed co-immunoprecipitation experiments. Carvedilol stimulation increased the amount of Gα_i_ bound to β_1_ARs in a dose-dependent manner, whereas it resulted in a decrease of Gα_i_ that could be co-immunoprecipitated with β_2_ARs (Fig. 3c, Supplementary Fig. 3a). As a control for the effect of detergent on protein interaction during the co-immunoprecipitation, experiments were also performed with 1% n-Dodecyl β-D-maltoside (DDM) lysis buffer and showed similar results (Supplementary Fig. 3b). The amount of Gα_i_ bound to β_2_ARs was increased by the balanced agonist isoproterenol (Fig. 3c), as we observed with the PLA experiments and again consistent with the previously identified process of Gα_s_/Gα_i_ switching^27^.

We next determined whether carvedilol could induce Gα_i_ protein activation using an antibody that specifically recognizes the active GTP-bound Gα_i_. Carvedilol stimulation promoted the activation of Gα_i_ in β_1_AR stable cells, but not in β_2_AR stable cells (Fig. 3d), which was blocked by PTX (Supplementary Fig. 3c).

**Fig. 4:**
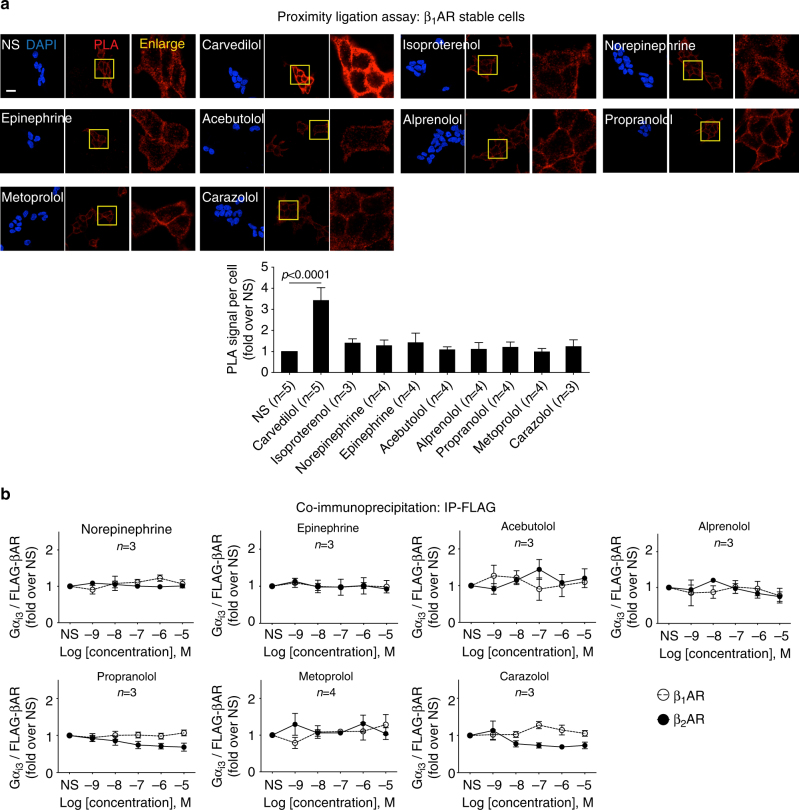
A number of βAR agonists or antagonists tested do not have significant effect on Gα_i_ recruitment. **a** β_1_AR stable cells were stimulated with vehicle or 10 μM indicated ligands for 5 min. Interaction of β_1_AR and Gα_i_ were detected by PLA. Scale bar = 20 μm. **b** β_1_AR or β_2_AR stable cells were stimulated with ligands at indicated concentration for 5 min. Gα_i_ recruitment was detected by co-immunoprecipitation. Both assays suggested that none of the ligands tested had similar effect of carvedilol on Gα_i_ recruitment. Data represent the mean ± SEM for *n* independent experiments as marked on the figure. Statistical significance vs. unstimulated cells was assessed using one-way ANOVA with Bonferroni correction

To determine whether Gα_i_ recruitment is specifically stimulated by carvedilol, we tested a number of βAR agonists and antagonists with PLA (Fig. 4a) and co-immunoprecipitation (Fig. 4b, Supplementary Fig. 4). Remarkably, no other ligand tested induced Gα_i_ recruitment to β_1_ARs, suggesting that this process may be a unique property of the β-arrestin-biased ligand carvedilol.

Collectively, these data support a concept that carvedilol selectively promotes the recruitment and activation of Gα_i_ to the β_1_AR subtype triggering β-arrestin-mediated signaling.

### Signaling dependence on both Gα_i_ and β-arrestins

**Fig. 5:**
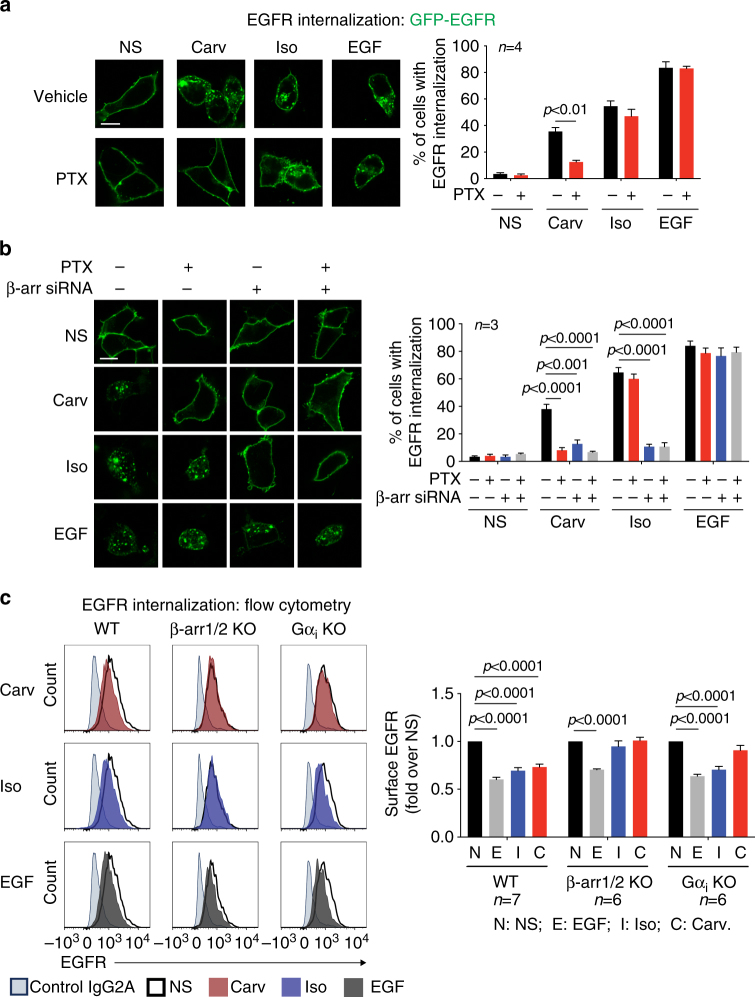
Both Gα_i_ and β-arrestins are required for carvedilol-induced β_1_AR-mediated EGFR internalization. **a** The effect of PTX on ligand-stimulated EGFR internalization. β_1_AR stable cells with transient transfection of GFP-EGFR were pretreated with vehicle or 200 ng per ml PTX for 16 h. Then the cells were stimulated with 10 μM carvedilol, 10 μM isoproterenol or 10 ng per ml EGF for 5 min. Both carvedilol and isoproterenol promoted EGFR internalization, but only the carvedilol-induced response was PTX sensitive. Scale bar = 10 μm. **b** Either PTX pretreatment or β-arrestins knockdown blocked carvedilol-induced EGFR internalization. β_1_AR stable cells were transfected with GFP-EGFR together with scrambled control siRNA or β-arrestin1/2 siRNA. 48 h after transfection, cells were pretreated with vehicle or 200 ng per ml PTX for 16 h before stimulation. Scale bar = 10 μm. **c** Carvedilol-induced EGFR internalization was abrogated in β-arrestins or Gα_i_ knockout cells. Wild type, β-arrestin1/2 knockout or Gα_i_ knockout cells were transfected with CFP-tagged β_1_ARs. Cells were stimulated with 10 μM carvedilol, 10 μM isoproterenol or 10 ng per ml EGF for 5 min. The EGFR level on cell surface was assessed by flow cytometry. Both carvedilol- and isoproterenol-induced EGFR internalization were impaired in the β-arrestin knockout cells, whereas only the carvedilol-induced response was blocked in the Gα_i_ knockout cells. Data represent the mean ± SEM for *n* independent experiments as marked on the figure. Statistical significance was assessed using two-tailed paired Student’s *t*-test (**a**) or one-way ANOVA with Bonferroni correction (**b**, **c**)

Previous studies have shown that carvedilol stimulation of β_1_ARs promotes the internalization and activation of EGFRs, which in turn activates downstream signaling such as ERK phosphorylation^10^. To dissect the mechanism of carvedilol-induced Gα_i_-dependent signaling, we tested the effect of PTX on β_1_AR-mediated EGFR internalization. We transfected HEK293 cells stably expressing β_1_ARs with GFP-tagged EGFR, and monitored internalization by confocal microscopy. When stimulated with isoproterenol or carvedilol, GFP-EGFR redistributed from the plasma membrane into endosomes, similar to that observed after EGF treatment (Fig. 5a). Pretreatment with PTX significantly blocked the carvedilol-induced EGFR internalization, while without any effect on the isoproterenol response, indicating a requirement for Gα_i_ for carvedilol-induced response (Fig. 5a).

Consistent with previous study showing that carvedilol-induced β_1_AR-mediated EGFR transactivation is β-arrestin-dependent^10^, siRNA knockdown of β-arrestin1 and β-arrestin2 abrogated both isoproterenol- and carvedilol-induced EGFR internalization (Fig. 5b, Supplementary Fig. 5a). While transactivation triggered EGFR internalization induced by both ligands are β-arrestin dependent, the precise molecular mechanism appears to have distinct features. Whereas the carvedilol-induced response requires both Gα_i_ and β-arrestin, the isoproterenol-induced response is PTX insensitive.

To more robustly determine the role of Gα_i_ and β-arrestin in carvedilol-stimulated EGFR transactivation, we generated β-arrestin or Gα_i_ deficient cells using CRISPR–Cas9 gene editing (Supplementary Fig. 5b). The wild type, Gα_i_ knockout or β-arrestin1/2 knockout cells were transfected with CFP-tagged β_1_AR. After ligand stimulation, the level of cell surface EGFRs was analyzed by flow cytometry (Fig. 5c). In wild-type cells, EGFRs were internalized following the treatment with EGF, isoproterenol or carvedilol. The depletion of Gα_i_ blocked carvedilol-induced EGFR internalization, whereas absence of Gα_i_ had no effect on EGF- or isoproterenol-induced responses. In contrast, β-arrestin1/2 knockout cells showed impaired EGFR internalization in response to both isoproterenol and carvedilol. Taken together, these results suggest that the carvedilol-induced EGFR internalization are dependent on both Gα_i_ and β-arrestins.

**Fig. 6:**
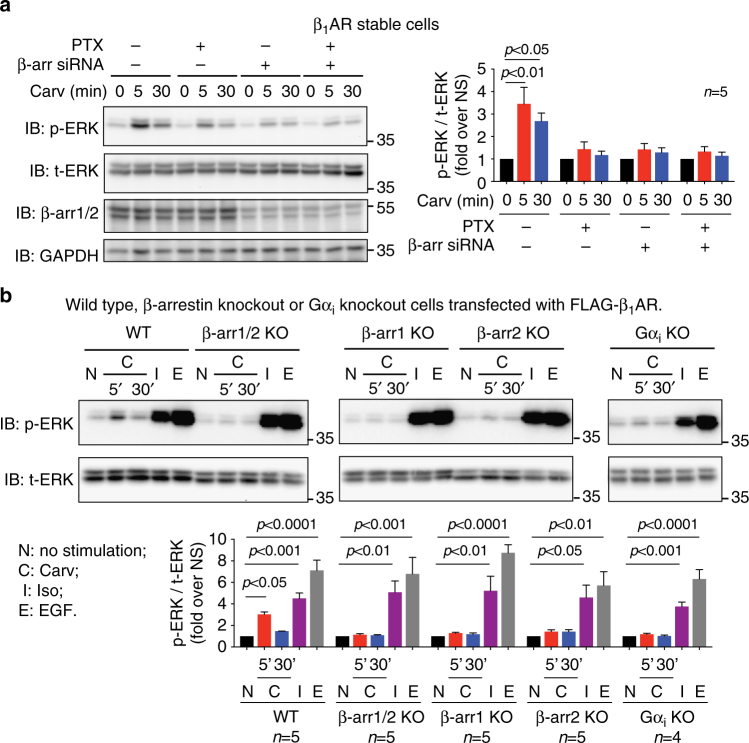
Carvedilol-induced β_1_AR-mediated ERK phosphorylation is dependent on both Gα_i_ and β-arrestins. **a** The effect of PTX and β-arrestin knockdown on carvedilol-stimulated ERK phosphorylation. β_1_AR stable cells with transfection of control siRNA or β-arrestin1/2 siRNA were pretreated with vehicle or PTX, then simulated with 10 μM carvedilol for 5 min or 30 min. Carvedilol-stimulated ERK phosphorylation was diminished by either PTX pretreatment or β-arrestins siRNA, suggesting the requirement of Gα_i_ and β-arrestins for this signaling. **b** The β_1_AR-mediated ERK phosphorylation in β-arrestin or Gα_i_ knockout cells. Wild type, β-arrestin knockout or Gα_i_ knockout HEK293 cells were transfected with FLAG-tagged β_1_ARs. Cells were stimulated with 10 μM carvedilol for 5 or 30 min, 10 μM isoproterenol or 10 ng per ml EGF for 5 min. The depletion of either β-arrestins or Gα_i_ impaired carvedilol-induced ERK phosphorylation. Data represent the mean ± SEM for *n* independent experiments as marked on the figure. Statistical significance vs. unstimulated cells was assessed using one-way ANOVA with Bonferroni correction

Consistent with our observation for EGFR internalization, carvedilol-induced ERK phosphorylation required both Gα_i_ and β-arrestins (Fig. 6a, b). Either Gα_i_ inhibition by PTX or β-arrestin knockdown with siRNA diminished carvedilol-induced ERK phosphorylation (Fig. 6a). Moreover, in HEK293 cells transfected with FLAG-β_1_ARs but depleted of either Gα_i_ or β-arrestin, carvedilol stimulated ERK activation was completely abrogated (Fig. 6b). Interestingly, removing either β-arrestin1 or β-arrestin2 prevented carvedilol-stimulated ERK phosphorylation, suggesting that both isoforms are required for carvedilol-stimulated signaling (Fig. 6b).

**Fig. 7:**
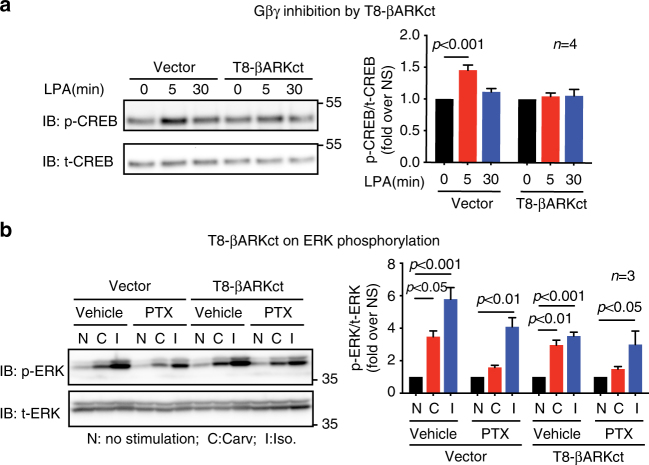
Gβγ subunits are not required for carvedilol-induced β_1_AR-mediated ERK phosphorylation. **a** Validation of the Gβγ inhibition by T8-βARKct. HEK293 cells with or without transient transfection of T8-βARKct were stimulated with 10 μM LPA for 5 min. T8-βARKct diminished the LPA-induced CREB phosphorylation, a known Gβγ-dependent process, confirming the inhibition of Gβγ subunits by T8-βARKct. **b** The Gβγ subunits are not required for carvedilol-induced β_1_AR-mediated ERK phosphorylation. β_1_AR stable cells with or without T8-βARKct transfection was pretreated with vehicle or 200 ng per ml PTX for 16 h. The cells were then stimulated with 10 μM carvedilol or 10 μM isoproterenol for 5 min. T8-βARKct did not have significant effect on ERK phosphorylation, suggesting that Gβγ subunits were not required. Data represent the mean ± SEM for *n* independent experiments as marked on the figure. Statistical significance vs. unstimulated cells was assessed using one-way ANOVA with Bonferroni correction

When βARs are stimulated by the balanced agonist isoproterenol, protein kinase A (PKA) activated by Gα_s_-dependent cyclic AMP phosphorylates the receptor leading to a switch of β_2_AR G protein coupling from Gα_s_ to Gα_i_. The now Gα_i_ coupled β_2_AR acts as a negative regulator of Gα_s_ signaling and activates ERK signaling via dissociated Gβγ subunits from heterotrimeric Gα_i_^27–29^. Here, we sought to determine if Gβγ subunits are required for carvedilol-stimulated Gα_i_-dependent ERK phosphorylation. Gβγ inhibition was achieved by transfection of T8-βARKct, a chimeric molecule consisting of two components: the C-terminus of the β adrenergic receptor kinase (βARKct) that competitively binds Gβγ, therefore acting as an inhibitor of Gβγ^30^; and the extracellular and transmembrane domain of CD8 receptor, which anchors the chimeric protein to the plasma membrane and potentiates its inhibitory effect^31^. The Gβγ blockade efficiency of T8-βARKct was confirmed by testing its effect on lysophosphatidic acid (LPA)-induced phosphorylation of cyclic AMP-responsive element-binding protein (CREB) (Fig. 7a). We show that the inhibition of Gβγ by T8-βARKct did not affect the carvedilol-induced ERK activation (Fig. 7b). This suggests that unlike isoproterenol stimulated Gα_i_-signaling achieved by G protein switching, carvedilol-induced Gα_i_-dependent β_1_AR signaling does not require Gβγ.

Collectively, these data demonstrate that both Gα_i_ and β-arrestins are required for carvedilol-induced β_1_AR signaling. Notably, either PTX pretreatment or β-arrestin knockdown was able to significantly block the carvedilol-induced β_1_AR-mediated EGFR internalization and ERK phosphorylation, and these responses were completely abrogated when either Gα_i_ or β-arrestin was depleted by gene editing. Taken together these data suggest that Gα_i_ and β-arrestins are likely involved in the same signaling cascade, rather than acting in parallel pathways downstream of β_1_AR.

### Phosphorylation of β_1_AR is not required for Gα_i_ recruitment

**Fig. 8:**
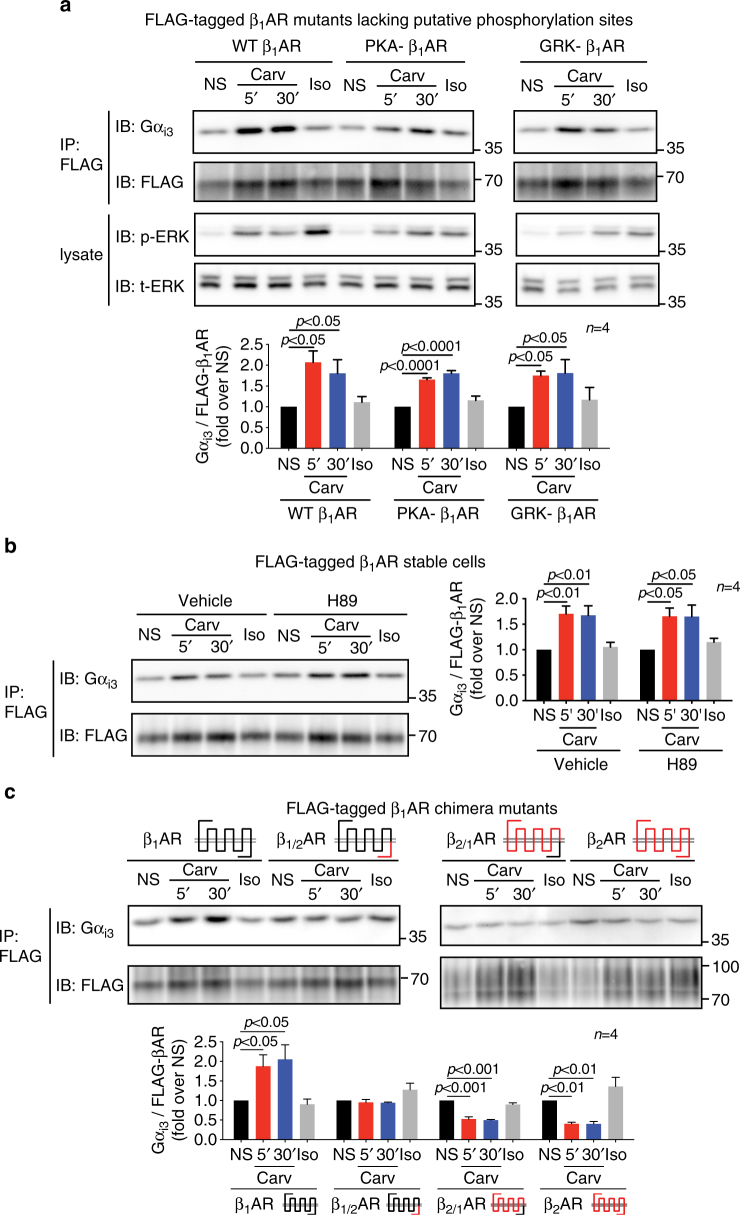
Neither PKA-mediated nor GRK-mediated β_1_AR phosphorylation is required for carvedilol-induced Gα_i_ recruitment. C-tail of β_1_AR is required but not sufficient for Gα_i_ recruitment. **a** HEK293 cells were transfected with FLAG-tagged wild-type, PKA- or GRK- β_1_ARs. Carvedilol promoted Gα_i_ recruitment to mutant β_1_ARs lacking the putative PKA- or GRK-mediated phosphorylation sites, to a similar extent as to the wild-type β_1_ARs. **b** HEK293 cells stably expressing FLAG-tagged β_1_ARs were pretreated with vehicle or 10 μM H89 for 30 min. The PKA inhibitor H89 did not have a significant effect on carvedilol-induced β_1_AR–Gα_i_ coupling. **c** HEK293 cells were transfected with FLAG-tagged β_1_AR, β_2_AR or chimeric βAR constructs in which the receptor C-tails were exchanged between the two receptor subtypes. Carvedilol did not promote Gα_i_ recruitment to the β_1_AR with C-tail from β_2_AR. On the other hand, the β_1_AR C-tail did not make β_2_AR capable of recruiting Gα_i_ with carvedilol stimulation. Data represent the mean ± SEM for n independent experiments as marked on the figure. Statistical significance vs. unstimulated cells was assessed using one-way ANOVA with Bonferroni correction

GRK-mediated receptor phosphorylation plays a critical role in β-arrestin-dependent signaling of βARs^32^. When β_1_ARs are stimulated by balanced agonists, such as isoproterenol or dobutamine, GRK-mediated β_1_AR phosphorylation of the carboxyl-terminal tail occurs and is required for agonist mediated β-arrestin recruitment^9, 33^. For the β_2_AR, a similar process has been show to occur whereby stimulation with the β-arrestin-biased agonist carvedilol promotes β_2_AR phosphorylation at specific GRK sensitive amino acid residues 355 and 356 of the c-terminal tail^21, 32^. Here, we sought to determine whether GRK-mediated phosphorylation of the β_1_AR is a necessary step in the carvedilol-induced Gα_i_ recruitment to the receptor. To address this question, we used a mutant β_1_AR that lacks the putative GRK phosphorylation sites within the receptor carboxyl-terminal tail (GRK-β_1_AR) and therefore unable to be phosphorylated by GRKs^9, 33^. We show that carvedilol stimulation increased Gα_i_ recruitment to a similar extent between wild type and GRK-β_1_ARs, as assessed by co-immunoprecipitation (Fig. 8a) and suggests that GRK-mediated β_1_AR phosphorylation is not required for carvedilol-induced Gα_i_ recruitment to the β_1_AR.

βARs can switch coupling from Gα_s_ to Gα_i_ when stimulated with a balanced agonist^27, 29^. In the Gα_s_-Gα_i_ switching model, agonist stimulated β_2_AR–Gα_i_ coupling is dependent on PKA-mediated receptor phosphorylation^27, 29^. To determine whether a similar mechanism is involved in the carvedilol-induced Gα_i_ recruitment to β_1_ARs, we used a β_1_AR mutant lacking the putative PKA phosphorylation sites (PKA-β_1_AR) or the PKA inhibitor H89. In our experiments, carvedilol stimulation promotes the Gα_i_ recruitment to PKA-β_1_ARs, similar as to wild-type receptors (Fig. 8a), whereas PKA inhibition with H89 did not have a significant effect (Fig. 8b).

Taken together, these data suggest that neither GRK- nor PKA-mediated receptor phosphorylation is required for carvedilol-induced Gα_i_ recruitment to β_1_ARs.

### β_1_AR C-tail is required but not sufficient for Gα_i_ coupling

Since the C-terminus of the βARs play vital roles in recruiting signal effectors and regulating downstream signaling^34^, we postulated that specific amino acid residues within the β_1_AR C-tail are critical for receptor subtype specificity of Gα_i_ recruitment. To test this hypothesis, we transfected HEK293 cells with βAR chimera mutants in which the C-tail of β_1_ARs was exchanged with that of β_2_ARs^14^, and assessed Gα_i_ recruitment to chimera βARs with co-immunoprecipitation. Carvedilol stimulation promoted the recruitment of Gα_i_ to the wild-type β_1_ARs, but was abrogated when the β_1_AR contained the C-tail from the β_2_AR (β_1/2_AR) (Fig. 8c). In contrast, the effect of carvedilol on Gα_i_ recruitment to the β_2_AR with the β_1_AR C-tail (β_2/1_AR) was similar to that of wild-type β_2_ARs. These data suggest that the C-tail of the β_1_AR is required for Gα_i_ recruitment, but alone is insufficient for this process to occur and is consistent with the crystal structure of the β_2_AR and G protein complex showing multiple receptor-G protein contact points^35^.

## Discussion

**Fig. 9:**
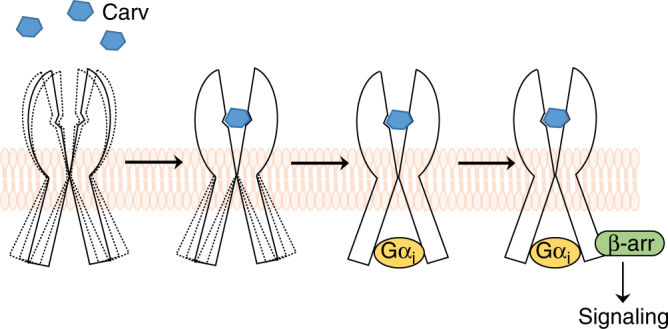
Schematic model of the carvedilol-induced Gα_i_-β-arrestin-biased signaling of β_1_ARs. Binding of carvedilol to the orthosteric site of the β_1_AR stablizes a distinct intermediate conformation that then promotes the recruitment of Gα_i_. The carvedilol- and Gα_i_-bound receptor in turn stabilizes a unique β_1_AR conformation that mediates β-arrestin-biased signaling

In this study, we provide new insight into the molecular mechanism of biased agonism at the β_1_AR. Carvedilol, a ligand classically known as a βAR antagonist, activates β-arrestin signaling by switching the uniquely Gα_s_-coupled β_1_AR to a Gα_i_-coupled receptor. We show that carvedilol is unique among a number of agonists and antagonists tested to promote the recruitment and activation of Gα_i_ to β_1_ARs. The recruited Gα_i_ in turn stabilizes a carvedilol-bound β_1_AR conformation that is required for β-arrestin-biased β_1_AR signaling as measured by EGFR internalization and ERK phosphorylation. These results indicate that the previously defined G protein bias vs. β-arrestin-bias may be attributed to ligand-induced selective coupling of receptors to specific G protein subtypes, i.e., G protein subtype bias. In our conceptual model for β_1_AR biased signaling, we speculate that binding of carvedilol to the β_1_AR stabilizes a unique receptor conformation that recruits and activates Gα_i_ to promote β-arrestin-mediated signaling (Fig. 9). While carvedilol is also known to stimulate β_2_AR signaling, Gα_i_ recruitment was not required for β_2_AR-mediated β-arrestin-biased signaling and suggests that different mechanisms for bias may be operative between βAR subtypes.

In the classical view of GPCR signaling, agonist stabilization of specific active conformational states promotes coupling of heterotrimeric G proteins and stimulation of downstream signaling^36^. Receptor signaling is then terminated by a process involving receptor phosphorylation, β-arrestin recruitment and receptor internalization. However, recent studies suggest that the classical “on–off” (active and inactive) model is oversimplified^20^, as GPCRs transmit signaling through multiple transducers to regulate diverse arrays of pathways. First, some GPCRs can couple to multiple G proteins. For example, the isoproterenol-activated β_2_AR switches coupling from Gα_s_ to Gα_i_^27^. In this study, we show that carvedilol switches the classical Gα_s_-coupled receptor β_1_AR to a Gα_i_-coupled receptor. However, in contrast to the Gα_s_-Gα_i_ switching of the β_2_AR, the carvedilol-induced β_1_AR–Gα_i_ coupling does not involve Gα_s_ activation and PKA-mediated receptor phosphorylation. The carvedilol-induced β_1_AR–Gα_i_ signaling is also different from the actions of classical Gα_i_-coupled receptors such as the muscarinic M_2_ receptor and the α_2_ adrenergic receptor^37^, as its activation of ERK is not mediated through Gβγ subunits. Second, in addition to their role as signal terminators for G protein signaling, β-arrestins can act as signal transducers in their own right. Current conceptual models support the idea that ligands may differentially stabilize distinct receptor conformations that recruit divergent portfolio of signaling transducers and effector proteins to active a select suite of cellular signaling pathways, a concept termed functional selectivity or biased agonism^15^.

The β-arrestin-biased ligand carvedilol has three unique features at the β_1_AR: (1) it has inverse efficacy for Gα_s_-dependent adenylyl cyclase activity; (2) it promotes the recruitment of Gα_i,_ not Gα_s_, to the β_1_AR; (3) it activates the classical β-arrestin signaling using a Gα_i_ paradigm. These unique signaling properties of carvedilol may be attributed to its ability to stabilize a distinct receptor active conformation^15^. For the β_2_AR, carvedilol uniquely induces significant conformational rearrangement around residue Lys263 and Cys265 in the third intracellular loop of the receptor, which may expose the loop toward intracellular surface and facilitate the receptor interaction with β-arrestins^15^. Though a previous study suggests the crystal structure of carvedilol-bound β_1_AR is similar to that of the cyanopindolol-bound inactive state structure^38^, additional conformations stabilized by carvedilol may require the binding of transducers such as Gα_i_ or β-arrestin. This requirement of transducer binding for receptor conformational stability is supported by the structural study of the β_2_AR showing that the interaction of a G protein, or a G protein-like-protein nanobody, is required to stabilize the agonist-induced receptor active conformation^39^. In our study, using a FRET-based β_1_AR conformation sensor, we show that carvedilol induces a change of FRET ratio, representing a receptor conformational change. Notably, the β_1_AR conformation induced by carvedilol is distinct from the one induced by the balanced agonist isoproterenol, as carvedilol increased the FRET ratio while isoproterenol decreased it. This further supports a concept that receptors can adopt distinct conformations when stimulated by different ligands. As our results show that carvedilol promotes the recruitment of Gα_i_ to β_1_ARs, while a wide range of other βAR ligands tested do not, it is possible that carvedilol induces a β_1_AR conformational change that exposes allosteric binding sites on the receptor to allow for receptor–Gα_i_ interaction. In turn, the bound Gα_i_ stabilizes the carvedilol-induced active receptor conformation and is consistent with our data where pretreatment with the Gα_i_ inhibitor PTX impairs the carvedilol activated β_1_AR conformation. Together these data support the concept that carvedilol-induced Gα_i_ is a positive allosteric modulator of the β-arrestin-biased β_1_AR active conformation.

While we have not determined the precise mechanism of how Gα_i_ binding to the carvedilol-occupied β_1_AR triggers β-arrestin signaling, we postulate that it may involve subsequent receptor phosphorylation in a process known as the “barcode” hypothesis^19^. Upon ligands stimulation, GPCRs can be phosphorylated by distinct GRK subtypes at specific sites. Previous study identified β_2_AR sites that are specifically phosphorylated by GRK2 and GRK6^32^. While the balanced agonist isoproterenol stimulates β_2_AR phosphorylation at both GRK2- and GRK6-specific sites, carvedilol only stimulates receptor phosphorylation at the GRK6-specific sites. This “barcode” phosphorylation pattern of receptors plays essential roles in regulating the recruitment and functionality of signaling transducers^19^. For instance, β_2_AR phosphorylation mediated by GRK2 and GRK6 induces distinct β-arrestin conformations, and differentially regulates receptor internalization and ERK activation^32^. Similarly for the β_1_AR, GRK2-mediated and GRK5/6-mediated receptor phosphorylation leads to distinct cellular responses^9, 40^, suggesting that a phosphorylation barcode for the β_1_AR may also direct β-arrestin signaling. To dissect the mechanism of how Gα_i_ regulates β_1_AR signaling, future studies will need to compare the isoproterenol- or carvedilol-induced barcode phosphorylation patterns of the β_1_AR, as well as the effect of Gα_i_ inhibitor PTX on it.

While our data show that both Gα_i_ and β-arrestins are required for carvedilol-induced biased signaling of the β_1_AR, whether β-arrestin is recruited to the carvedilol occupied β_1_AR remains to be determined. Using a number of methodologies, such as co-immunoprecipitation, confocal- or bioluminescence resonance energy transfer-based assays, we were unable to detect carvedilol-induced β-arrestin recruitment to the β_1_AR. This may be due to a number of reasons: (1) ligand-induced β-arrestin recruitment and activation is rapid, within 2 s after stimulation, and reversible^41^; (2) the affinity of the β_1_AR–β-arrestin interaction is low. Both the β_1_AR and the β_2_AR are known as class A receptors, since they are characterized by transient and weak interaction with β-arrestins along with a rapid recycling to the plasma membrane after internalization. To demonstrate carvedilol triggered β-arrestin recruitment to the β_2_AR, previous studies used a chimeric receptor consisting of the β_2_AR fused to vasopressin V_2_ receptor cytoplasmic tail (β_2_AR-V_2_R) to increase the affinity of β-arrestin to the ligand occupied receptor^21^. However, as we have shown (Fig. 8c), the C-tail of the β_1_AR is required for Gα_i_ recruitment. Therefore substituting the β_1_AR C-tail with the V_2_R tail would not provide a chimeric receptor suitable to study the role of Gα_i_ in carvedilol stimulated β-arrestin recruitment. Importantly, we cannot exclude that the carvedilol-stimulated β_1_AR signaling is mediated by β-arrestin by an indirect mechanism that does not require direct binding of β-arrestin to the β_1_AR. A recent study identified unique features for the β_1_AR with respect to β-arrestin interaction and activation^42^, where a brief interaction with the activated β_1_AR is sufficient to target β-arrestin2 to clathrin-coated structures and trigger ERK signaling even in the absence of receptor association^42^. This β-arrestin “activation at a distance” mechanism suggests that a β_1_AR–β-arrestin complex may not be essential for the activation of β-arrestin-dependent signaling and could explain our findings for a role of β-arrestin in carvedilol-induced signaling without a direct β_1_AR–β-arrestin interaction. Lastly, it is also possible that instead of directly engagement with the β_1_AR, β-arrestins could associate with other components of the signaling cascade such as the transactivated EGFR. This has recently been shown for the vasopressin V_2_ receptor signaling, where β-arrestins are recruited to, and act downstream of, the transactivated insulin-like growth factor receptor^43^.

Carvedilol is a βAR antagonist (β-blocker), a family of drugs that are widely used in the therapeutic treatment of cardiovascular diseases such as hypertension and heart failure, as β_1_ARs and β_2_ARs are predominant GPCR subtypes expressed in mammalian heart and play vital roles in the regulation of cardiac function^4^. In heart failure, treatment with β-blockers improves left ventricle function, reverses the pathological cardiac remodeling, and reduces mortality and morbidity^44, 45^. However, β-blockers have different clinical efficacies. Some evidence suggests that carvedilol has a superior effect on cardiovascular survival to other β-blockers^46^. The molecular basis for this remains to be elucidated, but has been attributed to the additional properties of carvedilol other than β-blockers, such as the antioxidant, antiproliferative effects and α_1_ adrenergic receptor blockade^47^. Interestingly, carvedilol appears to be unique among βAR blockers in that it can activate β-arrestin-dependent signaling that confers cardioprotection^10, 21^. Given the possible cardioprotective role of Gα_i_ during cardiac stress^48^ and the ability of carvedilol to promote β_1_AR–Gα_i_ coupling, it is possible that this unique property of carvedilol is also important for its therapeutic efficacy.

In conclusion, we identify a new signaling mechanism of GPCR biased agonism. To date, the β_1_AR was considered to be predominantly coupled to Gα_s_, and β-arrestin-dependent β_1_AR signaling to be independent of G proteins. However, our data supports a concept where carvedilol has three unique properties at the β_1_AR: (1) it is inert with respect to Gα_s_; (2) it recruits Gα_i_ and converts the β_1_AR from a Gα_s_-coupled receptor to one that couples to Gα_i_; and (3) it activates classical β-arrestin-dependent signaling in a Gα_i_ paradigm. These data suggest a greater complexity for receptor signaling bias than previously appreciated in that coupling of distinct G protein subtypes to the activated receptor are needed for β-arrestin-biased agonism. These data also have important implications when considering the development of new therapeutic ligands designed to selectively target β-arrestin-biased signaling pathways.

## Methods

### Cell culture

HEK293 cells (American Type Culture Collection) stably expressing FLAG-tagged β_1_AR or β_2_AR are maintained and transfected as previously described^33, 49^. Cells were periodically treated with BMCyclin (Roche) to avoid mycoplasma contamination. Cells were incubated overnight in serum-free medium supplemented with 0.1% BSA, 10 mM HEPES and 1% penicillin–streptomycin and pretreated with pertussis toxin (200 ng per ml, overnight), H89 (10 μM, 30 min) or propranolol (10 μM, 30 min) before ligand stimulation. HEK293 cells stably expressing β_1_AR-FRET sensor were used for the FRET experiments.

### Generation of β-arrestin or Gα_i_ knockout cell line

Plasmids carrying *S. pyogenes* Cas9 (SpCas9) next to a cloning site for guide RNA (gRNA) with EGFP (pSpCas9 (BB)-2A-GFP, Addgene 48138) or puromycin resistant gene (pSpCas9(BB)-2A-Puro, Addgene 48139) were obtained from Addgene (deposited by the laboratory of Dr. F. Zhang^50^). Designing of the guide RNAs for Gα_i_ or β-arrestins and cloning the guide RNAs into the Cas9 plasmids were performed as previously described^50^.

For β-arrestin knockout cells, β-arrestin1 was targeted using guide sequence oligos (top: CACCGCATCGACCTCGTGGACCCTG; bottom: AACCAGGGTCCACGAGGTCGATGC). β-arrestin2 was targeted using guide sequence oligos (top: CACCGCGTAGATCACCTGGACAAAG; bottom: AAACCTTTGTCCAGGTGATCTACGC). The guide sequence oligos were cloned into pSpCas9(BB)-2A-Puro. After confirming the cloning by sequencing, plasmids were transfected into HEK293 cells using Fugene 6 transfection reagent (Promega). 72 h after transfection, cells were harvested to check INDEL (insertion deletion) in the genome by surveyor’s assay. Puromycin (2.5 μg per ml) was added into the medium of surveyor positive cells to select cells with the plasmid containing puromycin resistant gene along with guide RNA and Cas9. The knockout of β-arrestins were confirmed by western blot.

For Gα_i_ knockout cells, Gα_i1_ was targeted using guide sequence oligos (top: CACCGCGCCGTCCTCACGGAGGTTG; bottom: AAACCAACCTCCGTGAGGACGGCGC), Gα_i2_ was targeted using guide sequence oligos (top: CACCGAGACAACCGCCCGGTACTGC, bottom: AAACGCAGTACCGGGCGGTTGTCTC), and Gα_i3_ was targeted using guide sequence oligos (top: CACCGGGACGGCTAAAGATTGACTT; bottom: AAACAAGTCAATCTTTAGCCGTCCC). The guide sequence oligos were cloned into pSpCas9 (BB)-2A-GFP. Plasmids targeting the three Gα_i_ subtypes were co-transfected into HEK293 cells. GFP positive cells were selected by fluorescence-activated cell sorting, diluted for growth and single cell colonies were obtained. The Gα_i_ knockout were confirmed by western blot.

### Immunoblotting and immunoprecipitation

Following stimulation, cells were scraped in 1% NP-40 lysis buffer (20 mM Tris, pH 7.4, 137 mM NaCl, 20% glycerol, 1% Nonidet P-40, 2 mM sodium orthovanadate, 1 mM PMSF, 10 mM sodium fluoride, 10 μg per ml aprotinin, 5 μg per ml leupeptin and phosphatase inhibitors) or 1% DDM lysis buffer (20 mM HEPES, 150 mM NaCl, 1% n-Dodecyl β-d-maltoside, protease inhibitors and phosphatase inhibitors). For immunoprecipitation of FLAG-tagged β_1_AR or β_2_AR, 1–2 mg of protein was incubated overnight with 30 μl of anti-FLAG M2 magnetic beads (Sigma). For immunoprecipitation of active Gα_i_, protein was incubated for 2 h with anti-active Gα_i_ antibody (New East Biosciences) and Protein A/G beads (EMD Millipore). Immunoprecipitates or cell lysate samples were separated by SDS-PAGE, transferred to PVDF membrane (Bio-Rad) and subjected to immunoblotting with various primary antibodies. Immunoblots were detected using enhanced chemiluminescence (Thermo Fisher Scientific) and analyzed with ImageJ software. Uncropped blots are shown in Supplementary Fig. 6.

### Antibodies

Please refer the information of antibodies to Supplementary Table 1.

### ERK phosphorylation in mice heart

Eight to 12-week-old gender-matched β_1_AR knockout (β_1_AR KO) mice and β_2_AR KO mice^51^ were used for this study. Three to six animals were used for each experimental group based on previous experiments. Randomization and blinding were not performed. Mice were pretreated with vehicle or 25 μg per kg pertussis toxin (PTX) via intraperitoneal injection. After 48 h, mice were anesthetized with ketamine (100 mg per kg) and xylazine (2.5 mg per kg) for 10 min. Heart was then excised and, with aorta cannulated to needle, perfused with perfusion buffer (118 mM NaCl, 4.7 mM KCl, 1.2 mM MgSO_4_, 1.2 mM KH_2_PO_4_, 2.5 mM CaCl_2_, 25 mM NaHCO_3_, 0.5 mM Na-EDTA, 5.5 mM glucose) with O_2_ bubbling through Langendorff apparatus (Hugo Sachs Harvard Apparatus) set at 37 °C. After 10 min perfusion, buffer was changed to perfusion buffer with vehicle or 10 μM carvedilol, and perfused for another 10 min. Heart was then removed from the system and left ventricle was excised and snap frozen in liquid nitrogen. Animal experiments carried out for this study were handled according to approved protocols and animal welfare regulations the Animal Care and Use Committee of Duke University Medical Center.

### Fluorescence resonance energy transfer measurement

FRET measurement was performed as previously described^26^. Briefly, HEK293 cells stably expressing β_1_AR-FRET sensor were cultured in glass-bottomed confocal dish. Cells were pretreated with vehicle or 200 ng per ml PTX for 16 h before experiment. On the day of experiment, cells were maintained in FRET buffer (10 mM HEPES, 0.2% BSA, 140 mM NaCl, 4.5 mM KCl, 2 mM CaCl_2_, 2 mM MgCl_2_, pH 7.4). FRET experiments were preformed using an Olympus IX-71 microscope. FRET was monitored as the emission ratio of YFP to Cerulean. Images were taken at 10 s interval and analyzed with ImageJ software.

### In situ proximity ligation assay

β_1_AR or β_2_AR stable cells were cultured in 35 mm poly-d-lysine coated glass-bottom confocal dish (MatTek). Following stimulation, cells were fixed in 4% paraformaldehyde for 15 min and permeabilized with 0.2% Triton-X-100 for 10 min. After blocked with blocking buffer from Duolink Detection Kit (Sigma) at 37 °C for 30 min, cells were incubated overnight at 4 °C with anti-β_1_AR (or β_2_AR) antibody from rabbit (Santa Cruz) in conjunction with anti-Gα_i_ antibody from mouse (New East Biosciences). The proximity ligation reaction was performed according to the manufacturer’s protocol using the Duolink Detection Kit (Sigma). Cells were mounted with DAPI Fluoromount-G (Southern Biotech). Images were recorded with Zeiss Axio Observer Z1 confocal microscope with ×40 objective. Data analysis was performed with ImageJ software. To quantify the mean PLA signal per cell, the red PLA fluorescence intensity was divided by the number of cells. The mean PLA signal of each data set was corrected by subtracting the background staining determined as the mean PLA signal of HEK293 cells without receptor overexpression. The relative fold over non-stimulation was normalized to the mean PLA signal of the unstimulated cells. In each experiment, 20–40 cells from three images were quantified for each condition.

### EGFR internalization assessed by confocal microscopy

HEK293 cells stably expressing FLAG-tagged β_1_AR were transfected with EGFR-GFP together with control siRNA or β-arrestin siRNA as described below. After 24 h, the transfected cells were plated into glass-bottomed confocal dish and kept in culture for additional 24 h. Following pretreatment with PTX and stimulation with ligands, cells were washed with ice-cold PBS and fixed with 4% paraformaldehyde for 15 min. EGFR internalization was visualized with Zeiss Axio Observer Z1 confocal microscope with ×63 objective. In each experiment, 50 cells of each condition were counted under microscope. The percentage of cells showing EGFR internalization was determined by the number of cells showing the intracellular aggregate of EGFR-GFP.

### EGFR internalization assessed by flow cytometry

HEK293 cells (wildtype, β-arrestin1/2 knockout or Gα_i_ knockout) were transfected with CFP-tagged β_1_AR. 48 h after transfection, cells were serum starved for 4 h before ligand stimulation. Following stimulation, cells were dissociated with accutase, washed with PBS and fixed in 4% formaldehyde for 15 min at room temperature. Fixed cells were enumerated, washed twice with staining buffer (PBS, 0.5% BSA, 2 mM EDTA) and blocked with 5% rat serum (Sigma) in staining buffer for 15 min. 1 × 10^6^ cells for each sample were stained with equal concentrations of either PE-conjugated EGFR antibody (R&D systems) or isotype control (R&D systems; PE-conjugated rat IgG2A) for 30 min at room temperature. Following staining, cells were washed twice with staining buffer and resuspended in PBS for analysis utilizing a BD LSRII flow cytometer (BD Biosciences). Data analysis was performed with FlowJo software. Following doublet exclusion, single cells were gated for CFP positivity. To quantify relative EGFR internalization following ligand stimulation, the following formula was utilized: geometric mean fluorescence intensity of the PE-EGFR signal for each data set minus MFI of the isotype control. The resultant value was normalized to the MFI of the unstimulated cells to assess the relative percentage of EGFR internalization.

### β-arrestin siRNA knockdown

SiRNAs targeting β-arrestin have been described previously^13^. A nonsilencing RNA duplex (5′-AAUUCUCCGAACGUGUCACGU-3′) was used as a control. HEK293 cells stably expressing FLAG-tagged β_1_AR were seeded into 10 cm dish on the day before to reach 30–40% confluence at the time of transfection. SiRNA were transfected using GeneSilencer Transfection Reagent (Genlantis) according to the manufacturer’s protocol. In brief, 20 μg siRNA and 240 μl siRNA dilution buffer were added into 180 μl serum-free medium, whereas 51 μl of transfection reagent was mixed with 300 μl serum-free medium. Both solutions were allowed to stand for 5 min at room temperature, then combined and incubated for additional 20 min. The mixture was then added to cells in the 10 cm dish with 4 ml serum-free medium. After 4 h incubation at 37 °C and 5% CO_2_, 5.5 ml of MEM containing 20% FBS and 2% penicillin–streptomycin were added into the dish. All assays were performed 3 d after siRNA transfection.

### Statistical analysis

Data are expressed as mean ± SEM. Statistical comparisons were performed using two-tailed Student’s *t*-test or ANOVA with Bonferroni correction for multiple comparisons in Graphpad Prism. Normality test was performed with Shapiro-Wilk test. Outlier data points more than two standard deviations from the mean were excluded from analysis. Differences were considered statistically significant at *P* < 0.05.

### Data availability

All data supporting the findings of this study are available from the authors upon request.

## Electronic supplementary material

Supplementary Information

Peer Review File
