# Supplementary material for: Gαi is required for carvedilol-induced β1 adrenergic receptor β-arrestin biased signaling
Source: Nat Commun. 2017 Nov 22;8:1706. doi: 10.1038/s41467-017-01855-z (PMC5700200; doi:10.1038/s41467-017-01855-z)
Supplement: Supplementary file 1 — Supplementary Information [file 41467_2017_1855_MOESM1_ESM.pdf]

Supplementary Figure 1

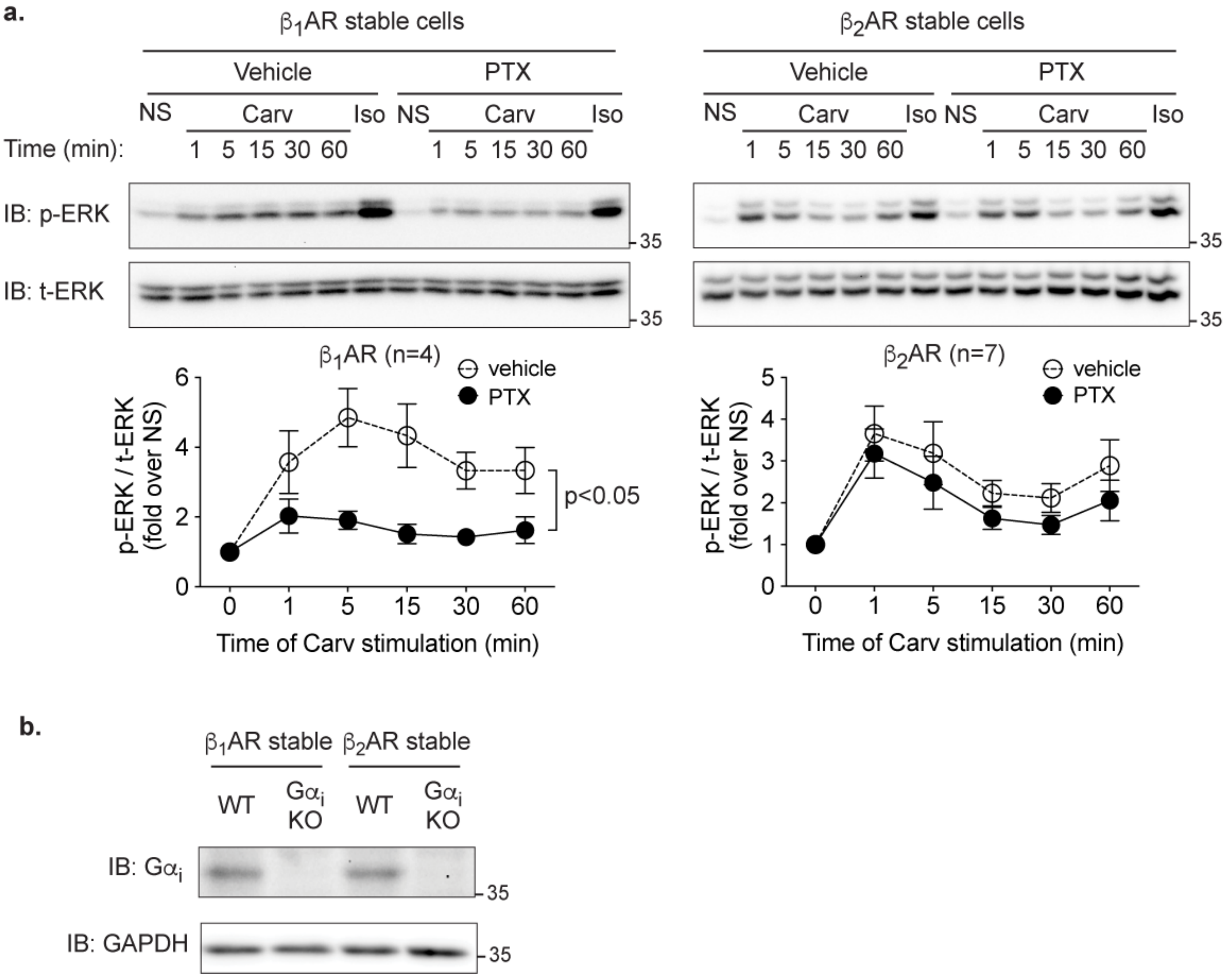

Supplementary Figure 1. $G\alpha_i$ inhibition by PTX or depletion by gene editing in ERK phosphorylation assay. (a) The effect of PTX on carvedilol-stimulated time course response of ERK phosphorylation. β_1AR or β_2AR stable cells were pretreated with vehicle or 200 ng per ml PTX for 16 h, then stimulated with 10 μM carvedilol for indicated time or with 10 μM isoproterenol for 5 min. Similar to the effect on the carvedilol dose response curve, PTX significant diminished carvedilol-induced ERK phosphorylation in β_1AR stable cells, but not β_2AR stable cells. Data represent the mean ± SEM for n independent experiments as marked on the figure. Statistical significance vs. control cells was assessed using two-way ANOVA with Bonferroni correction. (b) Representative blots showing $G\alpha_i$ depletion in β_1AR or β_2AR stable cells. All three subtypes ($G\alpha_{i1}$, $G\alpha_{i2}$, and $G\alpha_{i3}$) were knocked out with CRISPR-Cas9 gene editing.

Supplementary Figure 2

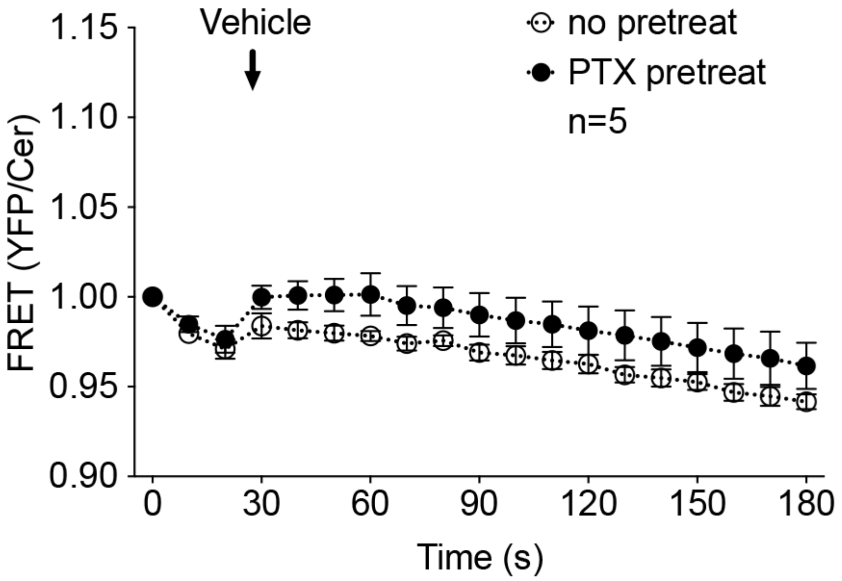

Supplementary Figure 2. The effect of PTX on the FRET-based β_1AR conformation sensor. HEK293 cells stably expressing the β_1AR-FRET sensor were pretreated with or without PTX. Vehicle without any ligands were added onto the cells while the FRET ratio was monitored. PTX alone did not alter the β_1AR conformational change. Data represent the mean ± SEM for n = 5 independent experiments. Statistical significance vs. unpretreated cells was assessed using two-way ANOVA with Bonferroni correction.

Supplementary Figure 3

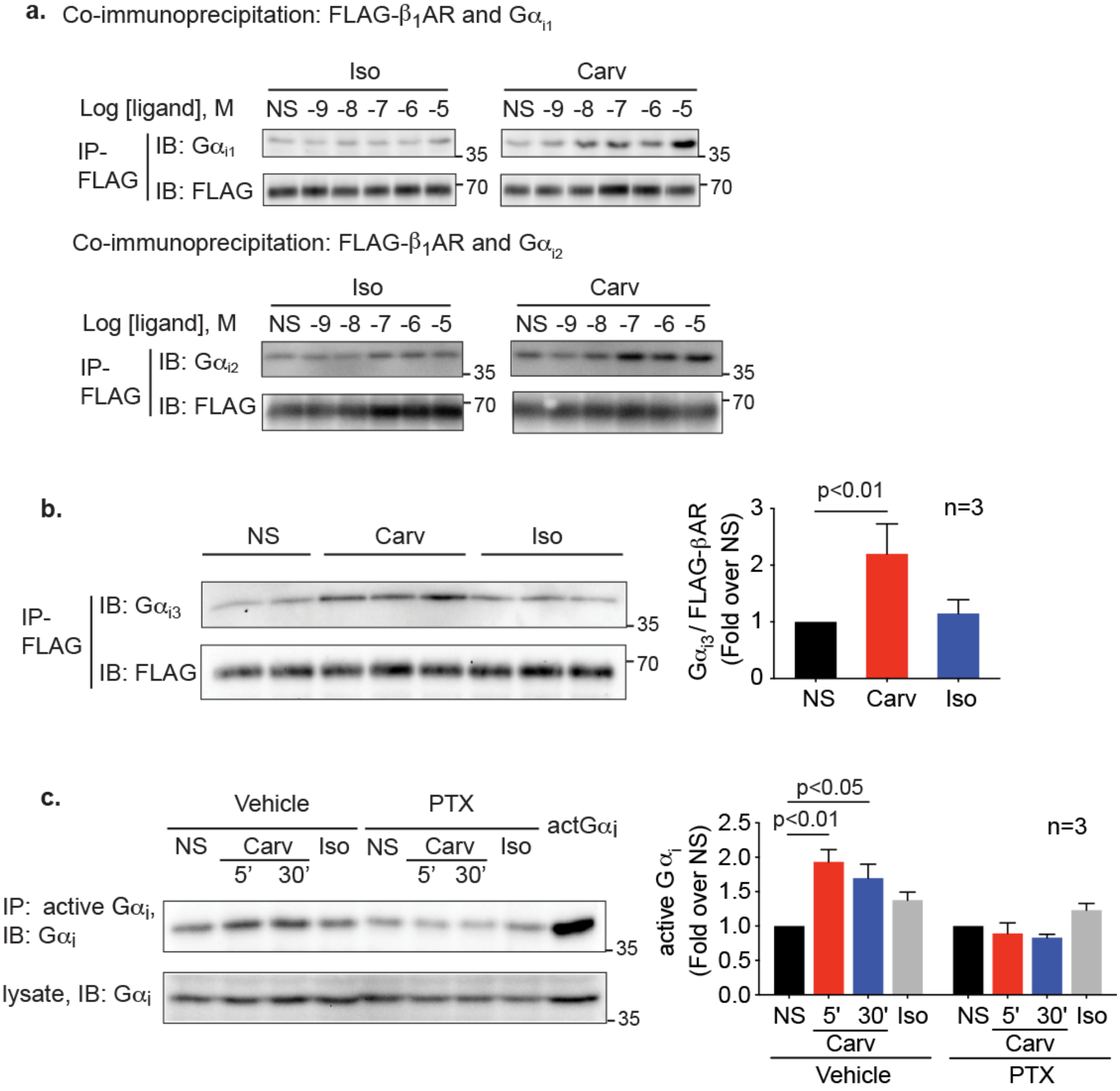

Supplementary Figure 3. Gα$_i$ recruitment and activation in β$_1$AR stable cells.
(a) Co-immunoprecipitation of Gα$_{i1}$ or Gα$_{i2}$ with β$_1$ARs. HEK293 cells stably expressing FLAG-tagged β$_1$ARs were stimulated with indicated concentration of carvedilol or isoproterenol for 5 min. β$_1$ARs were immunoprecipitated with anti-FLAG M2 beads, and bound Gα$_{i1}$ or Gα$_{i2}$ were detected with specific antibody by western blot. (b) Testing whether different detergent affects the β$_1$AR-Gα$_i$ coupling. Cells were lysed and co-immunoprecipitation was performed in 1% DDM lysis buffer. The change of detergent did not alter the carvedilol-promoted Gα$_i$ recruitment to β$_1$ARs. (c) As expected, PTX blocked Gα$_i$ activation in the carvedilol-stimulated β$_1$AR stable cells. Data represent the mean ± SEM for n independent experiments as marked on the figure. Statistical significance vs. unstimulated cells was assessed using one-way ANOVA with Bonferroni correction.

Supplementary Figure 4

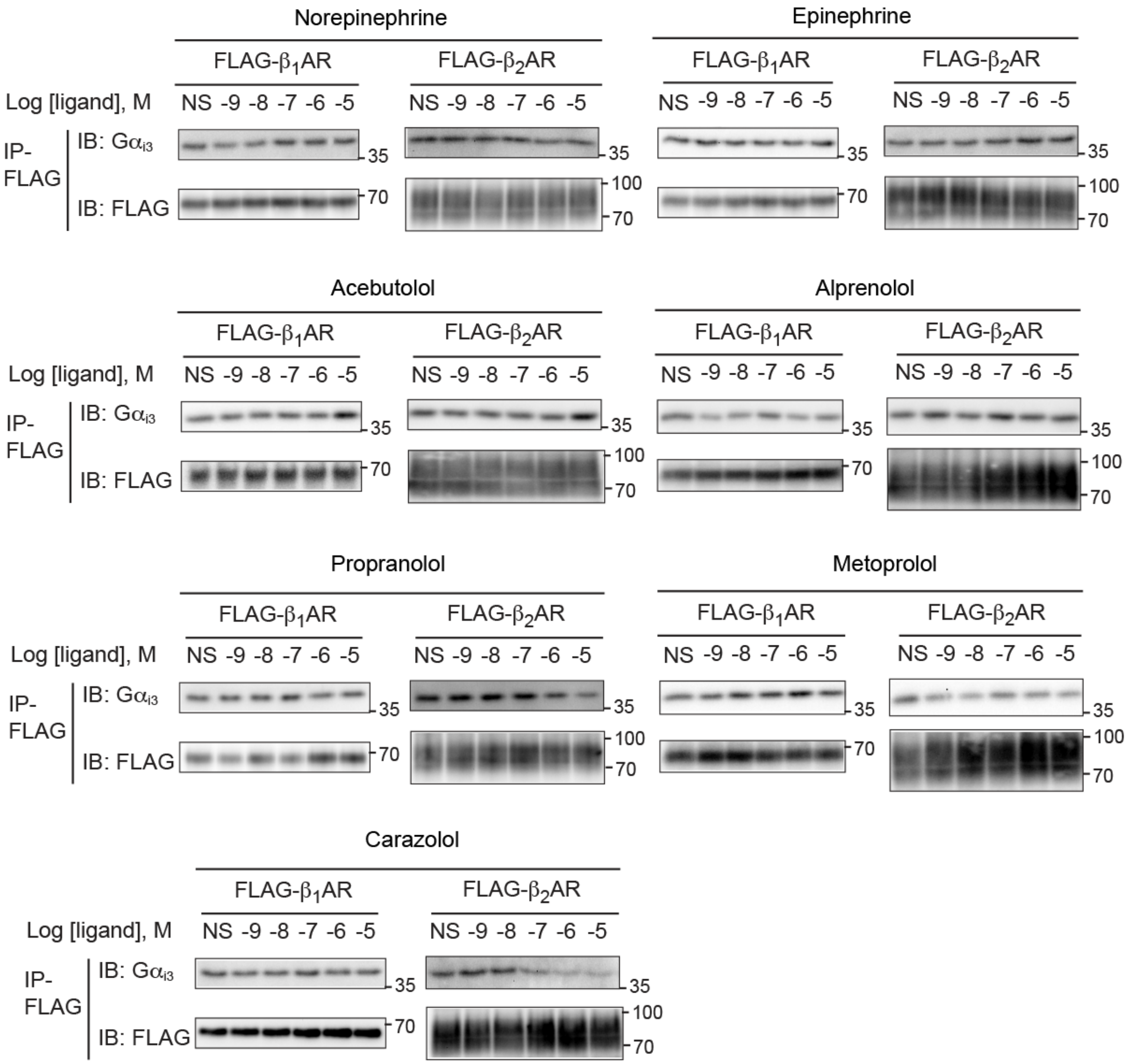

Supplementary Figure 4. Representative blots of Fig. 4b, showing the effect of βAR ligands on $G\alpha_i$ recruitment.

Supplementary Figure 5

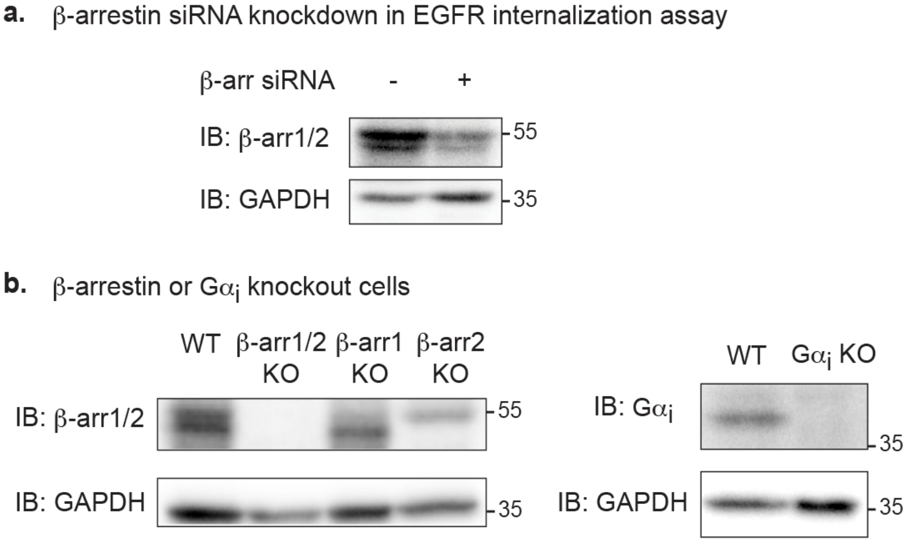

Supplementary Figure 5. Depletion of β-arrestins or $G\alpha_i$ in HEK293 cells.
(a) Representative blots showing the β-arrestin knockdown with siRNA in the EGFR internalization assay (Fig. 5b). The amount of both β-arrestin1 (upper band) and β-arrestin2 (lower band) are significantly decreased with in cells transfected with β-arrestin siRNAs.
(b) Representative blots showing the depletion of β-arrestins or $G\alpha_i$ in HEK293 cells. The β-arrestin1/2 double knockout, β-arrestin1 or β-arrestin2 single knockout, and the $G\alpha_i$ knockout cells were generated with CRISPR-Cas9 gene editing.

Supplementary Figure 6

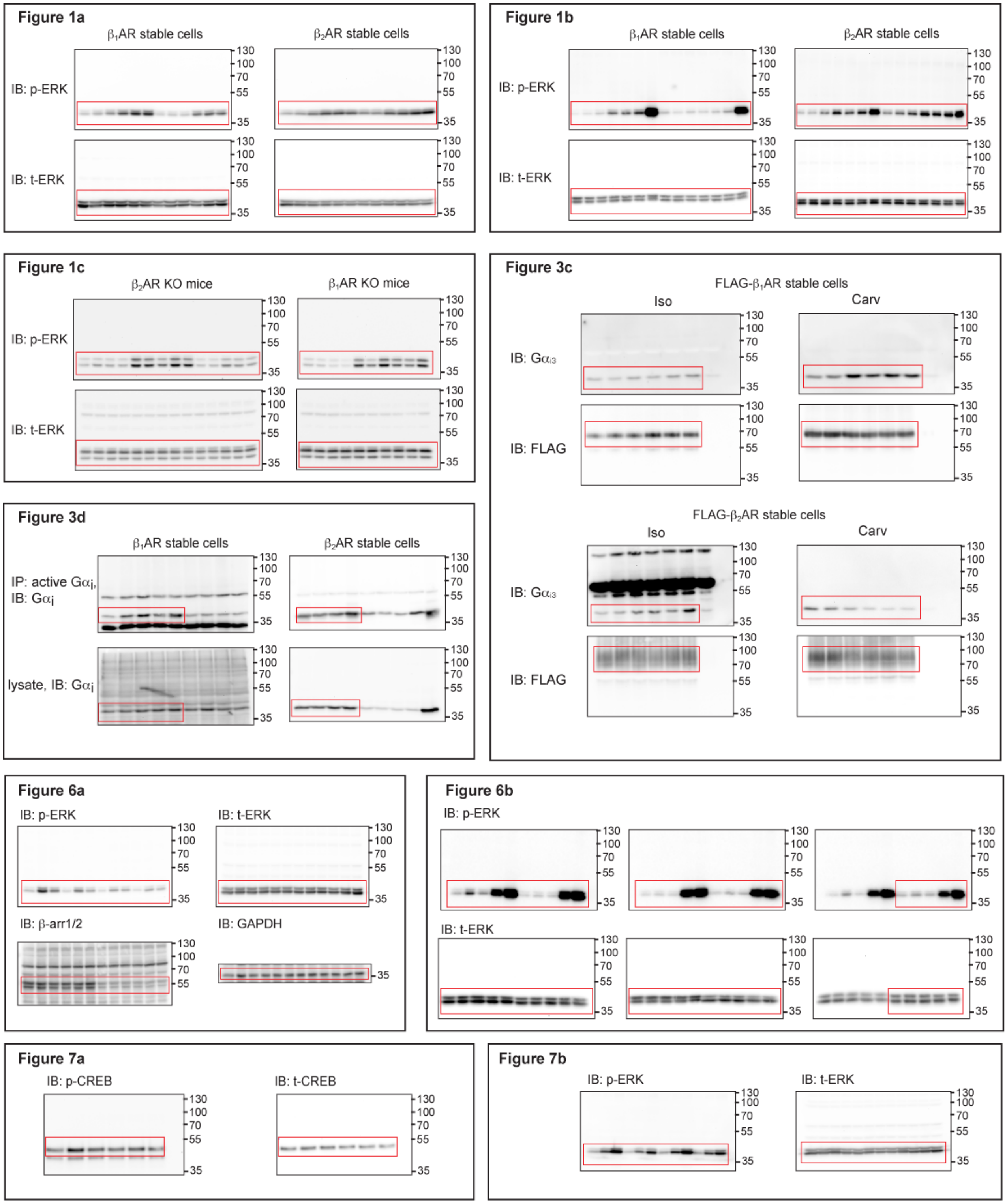

Supplementary Figure 6 - continued

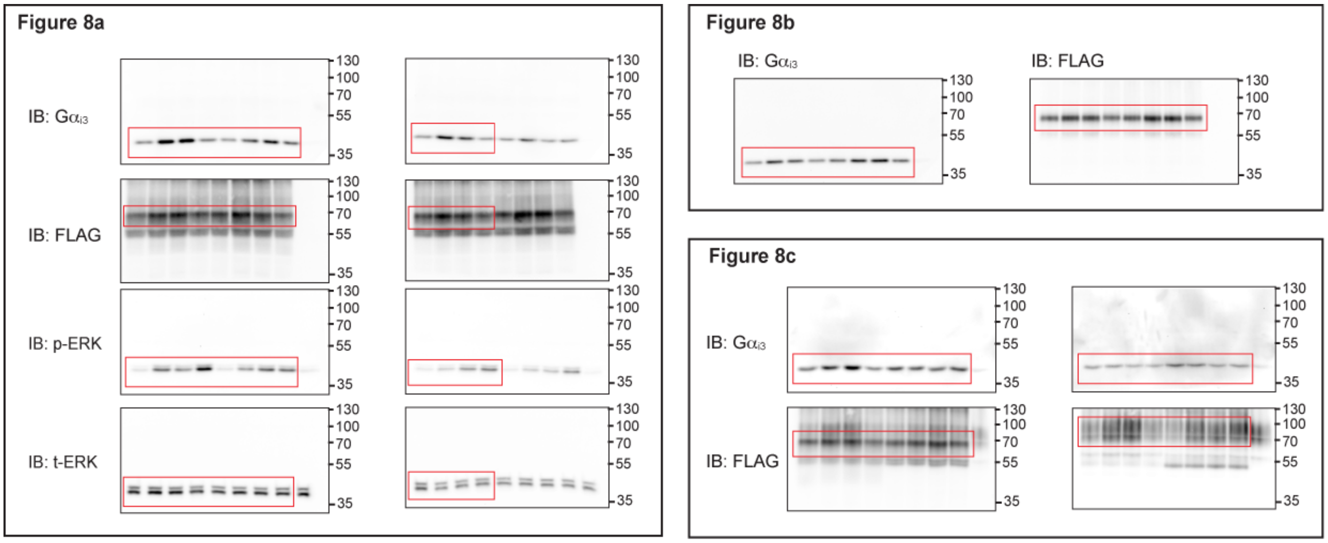

Supplementary Figure 6. Uncropped blots.

Supplementary Table 1

Antibody	Species	Catalog#	Source	Western blot	Immuno-staining	Immuno-precipitation	Flow cytometry
phospho-ERK	rabbit	9101	Cell Signaling	1:1000			
total-ERK	rabbit	06-182	EMD Millipore	1:3000			
β_1AR	rabbit	SC-568	Santa Cruz		1:500		
β_2AR	rabbit	SC-569	Santa Cruz		1:500		
$G\alpha_i$	mouse	26003	NewEast Biosciences	1:1000	1:200		
$G\alpha_{i3}$	rabbit	SC-262	Santa Cruz	1:500			
$G\alpha_{i1}$	rabbit	SC-391	Santa Cruz	1:500			
$G\alpha_{i2}$	rabbit	SC-7276	Santa Cruz	1:500			
FLAG	rabbit	F2555	Sigma Aldrich	1:1000			
active $G\alpha_i$	mouse	26901	NewEast Biosciences			1 μg for 1 mg total protein	
PE-conjugated EGFR	rat	FAB10951P	R&D system				1:5
PE-conjugated rat IgG2A	rat	IC006P	R&D system				1:5
β-arrestin1/2 (mix of A1CT and A2CT)	rabbit		Gift from Dr. R.J. Lefkowitz	1:3000			
phospho-CREB	mouse	9196	Cell Signaling	1:1000			
total-CREB	rabbit	9197	Cell Signaling	1:1000			

Supplementary Table 1. Antibodies.
